# Inflammatory Markers for Arterial Stiffness in Cardiovascular Diseases

**DOI:** 10.3389/fimmu.2017.01058

**Published:** 2017-08-31

**Authors:** Ioana Mozos, Clemens Malainer, Jarosław Horbańczuk, Cristina Gug, Dana Stoian, Constantin Tudor Luca, Atanas G. Atanasov

**Affiliations:** ^1^Department of Functional Sciences, “Victor Babes” University of Medicine and Pharmacy, Timisoara, Romania; ^2^Center for Translational Research and Systems Medicine, “Victor Babes” University of Medicine and Pharmacy, Timisoara, Romania; ^3^Independent Researcher, Vienna, Austria; ^4^The Institute of Genetics and Animal Breeding, Polish Academy of Sciences, Jastrzębiec, Poland; ^5^Department of Microscopic Morphology, “Victor Babes” University of Medicine and Pharmacy, Timisoara, Romania; ^6^2nd Department of Internal Medicine, “Victor Babes” University of Medicine and Pharmacy, Timisoara, Romania; ^7^Department of Cardiology, “Victor Babes” University of Medicine and Pharmacy, Timisoara, Romania; ^8^Department of Pharmacognosy, Faculty of Life Sciences, University of Vienna, Vienna, Austria; ^9^Department of Vascular Biology and Thrombosis Research, Center for Physiology and Pharmacology, Medical University of Vienna, Vienna, Austria

**Keywords:** inflammatory markers, arterial stiffness, inflammation, cardiovascular diseases, cardiovascular risk factors

## Abstract

Arterial stiffness predicts an increased risk of cardiovascular events. Inflammation plays a major role in large arteries stiffening, related to atherosclerosis, arteriosclerosis, endothelial dysfunction, smooth muscle cell migration, vascular calcification, increased activity of metalloproteinases, extracellular matrix degradation, oxidative stress, elastolysis, and degradation of collagen. The present paper reviews main mechanisms explaining the crosstalk between inflammation and arterial stiffness and the most common inflammatory markers associated with increased arterial stiffness, considering the most recent clinical and experimental studies. Diverse studies revealed significant correlations between the severity of arterial stiffness and inflammatory markers, such as white blood cell count, neutrophil/lymphocyte ratio, adhesion molecules, fibrinogen, C-reactive protein, cytokines, microRNAs, and cyclooxygenase-2, in patients with a broad variety of diseases, such as metabolic syndrome, diabetes, coronary heart disease, peripheral arterial disease, malignant and rheumatic disorders, polycystic kidney disease, renal transplant, familial Mediterranean fever, and oral infections, and in women with preeclampsia or after menopause. There is strong evidence that inflammation plays an important and, at least, partly reversible role in the development of arterial stiffness, and inflammatory markers may be useful additional tools in the assessment of the cardiovascular risk in clinical practice. Combined assessment of arterial stiffness and inflammatory markers may improve non-invasive assessment of cardiovascular risk, enabling selection of high-risk patients for prophylactic treatment or more regular medical examination. Development of future destiffening therapies may target pro-inflammatory mechanisms.

## Introduction

The elasticity and distensibility of arteries maintain a relatively constant blood pressure, despite the pulsating nature of the blood flow by every heartbeat. Arteries expand by receiving blood ejected from the heart during systole and expel it to the periphery during diastole to supply the peripheral circulation with a steady flow of blood during both cardiac cycles (1). However, as a hallmark of normal aging and apart from that also in association with many diseases compliance and distensibility of arteries decrease and the term “arterial stiffness” is used to qualitatively indicate these decreased elastic vessel wall properties (2). An increased arterial stiffness leads to a decreased buffer capacity of the arteries and an increase in pulse pressure (PP) and pulse wave velocity (PWV), causing an early return of the reflected waves and thereby an augmentation of late systolic pressure (3). As a consequence, the left ventricle has to generate an extra workload to overcome the augmented pressure, which is associated with an increased demand of oxygen and in the long-term development of left ventricular hypertrophy and heart failure (4). Insufficient arterial compliance furthermore transmits the increased pulsatile pressure deeper into the periphery and damages microvasculature of distal-end organ systems, especially in the kidney and the brain (5).

Arterial stiffness is considered a growing epidemic, associated with an increased risk of cardiovascular events (6–8). It is an early sign of structural and functional changes of the vessel wall and an independent predictor of cardiovascular disorders, that arise as a consequence of arteriosclerosis and atherosclerosis (9, 10). Several other chronic disorders may also contribute to an increase in arterial stiffness. The most important vascular changes related to increased arterial stiffness are vascular fibrosis due to collagen deposition, fragmentation of elastic fibers (elastolysis), crosslinking of collagen and elastin fibers by advanced glycation end products, and extensive vessel wall calcification (11–14). PWV and augmentation indices are commonly used measures of arterial stiffness and wave reflection.

Local inflammation is a complex non-specific protective response of vascular tissue to injury in order to eliminate its cause. Atherosclerosis is considered an inflammatory disease, because low-grade inflammation contributes to all phases of atherosclerosis development, starting with the initial phase of endothelial dysfunction, up to the formation of the mature atherosclerotic plaque, disruption of atherosclerotic injuries, precipitation of plaque rupture, and the acute thrombotic complications of atheroma (11, 15–19). Increased levels of inflammatory markers, e.g., erythrocyte sedimentation rate (ESR), C-reactive protein (CRP), and interleukin-6 (IL-6) have been associated with cardiovascular mortality and morbidity (19). As induction of experimental inflammation increases arterial stiffness, a cause–effect relationship between the two can be established, and anti-inflammatory therapy may, at least in part, reduce arterial stiffness (20, 21).

Both markers of inflammation and arterial stiffness are predictors of cardiovascular events (19). Understanding the mechanisms linking inflammation and arterial stiffness enables to apply a suitable anti-inflammatory therapy in order to reduce cardiovascular risk in several conditions.

Also, systemic inflammation has been shown to increase the risk of cardiovascular disease by accelerating atherosclerosis, destabilizing plaques, impairing endothelial function, or causing premature arterial stiffness (21–23). Considering that arterial stiffness is often the result of integrating the damage of cardiovascular risk factors on the arterial wall over a long period of time, prophylactic cardiovascular measures may open a window of opportunity to prevent the occurrence of cardiovascular disease even before first symptoms become immanent, which is an important aspect of health care in the ever-aging Western societies (10).

It is the aim of the present paper to highlight the role of inflammation on large vessels in view of arterial stiffness, review the main mechanisms explaining the crosstalk between inflammation and arterial stiffness and the most common inflammatory markers associated with an increased arterial stiffness, considering the most recent clinical and experimental studies published in biomedical research. Understanding the role of inflammation in the pathophysiology of arterial stiffness is crucial in order to enable development of new therapies by modulation of inflammatory pathways, which have been identified as major targets for the treatment of arterial stiffness.

## Mechanisms Underlying the Link Between Inflammation and Arterial Stiffness

Cardiovascular risk factors induce a state of inflammation able to impair vascular function (15). Activation of vascular smooth muscle cells (VSMCs) due to cardiovascular risk factors increases synthesis of the extracellular matrix and enables their migration from the vascular media to the intima (24).

Atherosclerosis is a syndrome caused due to chronic inflammatory interactions of white blood cells (WBCs) in the wall of arteries, and therefore, plasma inflammatory markers are considered potential tools for cardiovascular risk prediction (15, 25, 26). The mechanisms underlying the association between inflammation and atherosclerosis are generally complex and multifaceted (17). An increase in circulating inflammatory mediators enables WBC infiltration into arteries (27). Macrophage activation is associated with the release of inflammatory cytokines and reactive oxygen species to amplify the inflammatory reaction, and after transformation into foam cells, they undergo necrosis, further release inflammatory stimuli, and are thereby creating the necrotic core of advanced lesions (28). The onset of the inflammatory cascade, in acute and chronic inflammatory diseases and systemic subclinical low-grade inflammation, *impairs endothelial function* and the mechanical properties of the arteries (17). Endothelial cells (ECs), through reduction of bioavailability of nitric oxide (NO) and an increase of endothelin-1 due to inflammation, contribute to arterial stiffening and progressing arterial stiffing, in turn, further impairs endothelial function, thus inducing a vicious cycle (1, 3, 29). Decreased NO also promotes leukocyte adhesion (15). Additionally, endothelial dysfunction is also associated with activation of ECs which become pro-inflammatory, increasing expression of adhesion molecules, produce monocyte chemoattractant protein-1 (MCP-1) and leukocyte transmigration and activation, involving cytokines (13, 15).

Antigen-presenting cells such as dendritic cells and effector T lymphocytes play an important role in the synthesis of proatherogenic cytokines, such as IL-2, IL-18, and interferon gamma, and are therefore also important in atherosclerotic plaque progression (30). A special role among proatherogenic cytokines has been attributed to IL-12 as its absence was shown to inhibit early, but not late, lesion development (30). In conclusion, activated endothelium contributes to the initiation and the perpetuation of vascular wall inflammation (17).

Furthermore, monocytes are attracted and recruited to the vessel wall, where they differentiate into macrophages and ingest oxidized LDL (oxLDL) *via* scavenger receptors or lectin-like oxidized low-density lipoprotein receptor 1 (LOX-1). In this context, it has been shown that overexpression of SIRT1 is able to decrease LOX-1 expression and prevent foam cell formation in a mouse model of atherogenesis regardless of serum lipid levels. The underlying mechanism for this effect was shown to rely on suppression of NFκB-signaling by deacetylating RelA/p65, thereby reducing LOX-1 expression and diminishing uptake of oxLDL and foam cell formation and consequently also arterial stiffness (31). Therefore, pharmacological activation of SIRT1 may also offer an attractive approach in the treatment of arterial stiffness. Exposure to several pro-inflammatory and proatherogenic stimuli has been shown *in vivo* to upregulate LOX-1 expression, which is the main oxLDL receptor (32), and also in humans, LOX-1 gene polymorphisms are associated with increased susceptibility to cardiovascular disease (33).

Vascular inflammation increases arterial stiffness also by *enabling vascular fibrosis and smooth muscle cell proliferation* (19). The cellular components of the arterial wall, the VSMCs, and ECs are involved in maintaining homeostatic balance of the arterial blood pressure (3). In addition, VSMC can also undergo transdifferentiation into an osteoblastic phenotype under inflammatory conditions enabling mineralization (phosphate uptake), and *calcium deposition* in the arterial media (3, 27). Inflammatory cells produce mediators such as cytokines and metalloproteinases that regulate cardiovascular remodeling (34). Activation of matrix metalloproteinases (MMPs), mediated by increased inflammatory markers, enable *degradation of elastin and collagen* of the vessel wall (18). Inflammation enables also *plaque rupture* and is thus further contributing to the occurrence of acute coronary syndromes (15).

Arteriosclerosis is the age-associated stiffening and dilation of arteries, accompanied by low-grade inflammation (35). Interestingly, in humans early signs of arterial aging (so called “fatty streaks”) are already observable in children in their first decade, but it usually takes several decades for progression into a symptomatic disease (36). Arterial aging has been identified as a key mechanism enabling development and progression of cardiovascular and other chronic disorders and is heavily influenced by lifestyle factors (37). The chronic pro-inflammatory profile within aging arteries is characterized by impaired angiotensin II (AII), mineralocorticoid receptor, and endothelin-1 signaling with the result of increased activity and/or expression of downstream pro-inflammatory transcription factors, whereas levels/activity of protective factors become reduced (38). Also, the process of cellular senescence is suggested to be an important contributor to immunosenescence and “*inflamm-aging*” since senescent cells acquire a secretory phenotype, characterized by enhanced secretion of inflammatory modulators, such as monocyte chemoattractant protein-1 (MCP-1, which attracts invasion of smooth muscle cells), and cytokines, such as IL-1, IL-6, and IL-17 (39). Pro-inflammatory cytokines enable platelet and endothelial activation, which is related to an increased risk of cardiovascular events (35). Vascular aging is also associated with an increased vascular smooth muscle tone, an increased activity of the renin-angiotensin-aldosterone system, and an increase of oxidative stress, all of which contributes to arterial proinflammation and age-related arterial remodeling (13, 38). Further contributors to vascular aging are elevated levels of MMPs, calpain-1 (facilitating calcification), transforming growth factor beta-1 (increasing the production of extracellular matrix), amyloid deposition as medin, accumulation of fibronectin and cell adhesion proteins, increase of pro-inflammatory transcription factors as well as increased synthesis of advanced glycation end products, and decreased arterial expression and activity of sirtuins (13, 38). The loss of balance between oxidative and antioxidative systems results in increased production of reactive oxygen species leading to inactivation of NO and increased nitrosative stress which contributes to age-associated *endothelial dysfunction* (13, 40). The *increased degradation of elastin fibers* is mediated by activation of MMPs and serine proteinases, accompanied by a decreased activity of its endogenous inhibitor TIMP-2 (tissue inhibitor of metalloproteinases 2) (18). MMP is involved in the occurrence of *uncoiled, stiffer collagen* due to its collagenolytic activity and degradation of the basement membranes (6, 18). The increased MMPs activity is mediated by increased activity of cell adhesion molecules and cytokines (41, 42). Increased VSMC migration, proliferation and senescence, extracellular matrix deposition, matrix calcification, amyloidization and glycation, and elastin fracture disrupt the endothelium and thus, foster vascular aging (38).

Systemic subclinical low-grade inflammation is closely related to most cardiovascular risk factors, especially to hypertension and diabetes, to all stages of atherosclerosis, to arteriosclerosis and to impaired arterial elastic properties (17, 43, 44). Reduction in inflammation can decrease arterial stiffness, which was demonstrated among others in patients with rheumatoid arthritis (RA) undergoing therapy with anti-tumor necrosis factor-α (TNF-α) agents (21). Also, statins and other cholesterol-reducing agents are reported in numerous studies to have beneficial effects on wave reflection and aortic stiffness reduction in several patient groups (45, 46).

**Figure 1:**
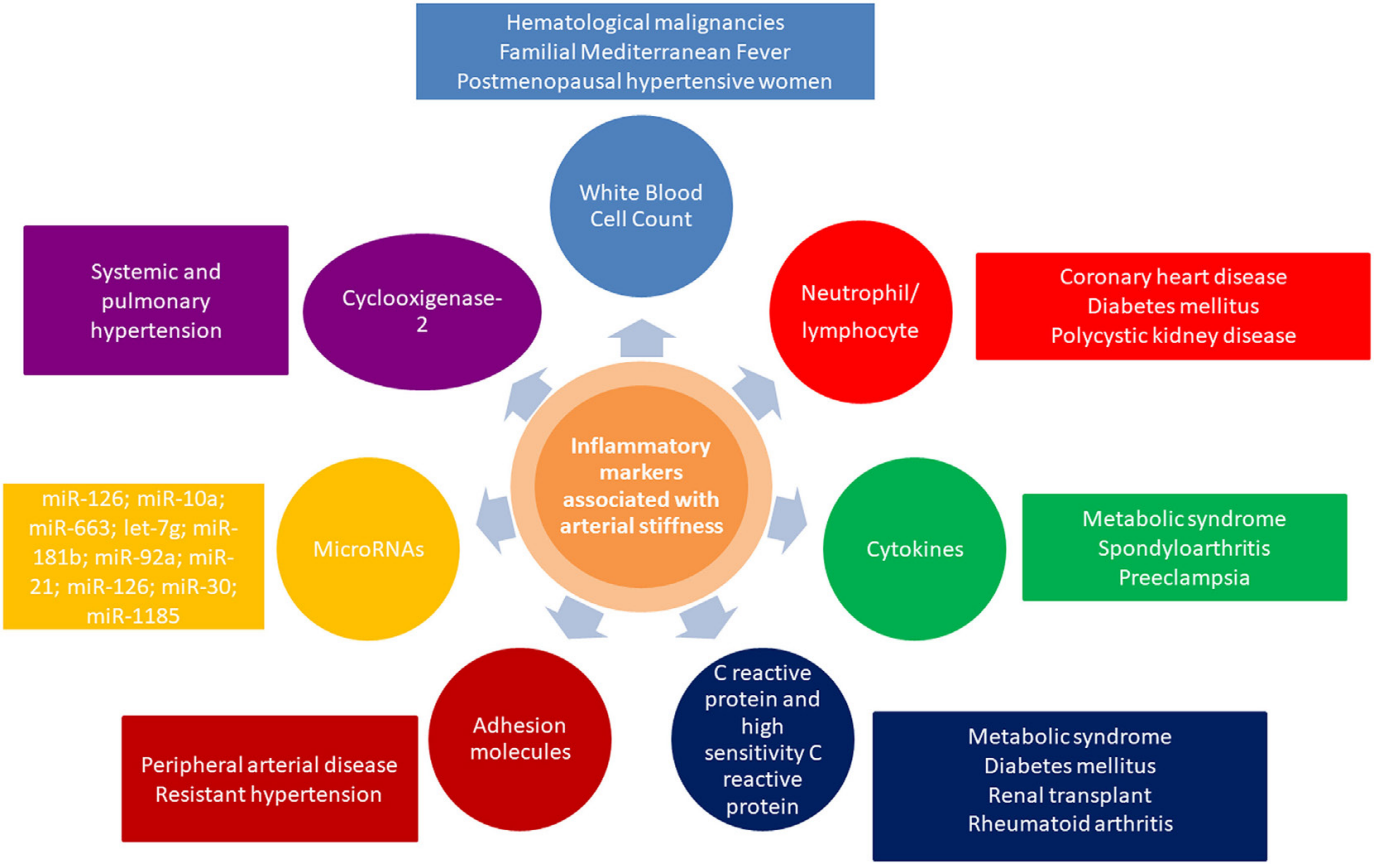
Overview of inflammatory markers associated with arterial stiffness and their crosstalk with other related conditions.

Pulse wave velocity inversely correlates with arterial distensibility, and an impaired arterial distensibility alters blood pressure, flow dynamics, increases afterload, and impairs coronary perfusion (27). Concluding, inflammation plays a major role in large artery stiffening, in the context of cardiovascular risk factors, atherosclerosis, arteriosclerosis, endothelial dysfunction, smooth muscle cell migration, vascular calcification, increased activity of metalloproteinases, extracellular matrix degradation, oxidative stress, elastolysis, degradation of collagen, and occurrence of uncoiled, stiffer collagen. Overview of relevant inflammatory markers associated with arterial stiffness and their crosstalk with other related conditions is presented in Figure 1.

## WBCs and Arterial Stiffness

An increased WBC has been associated with arterial stiffness and other atherosclerotic events in multiple studies (47). Chronic low-grade inflammation in the arterial wall may play an important role in the initiation and progression of cardiovascular diseases, considering that stimulated WBCs adhere to the vascular endothelium and easily penetrate the intima, causing capillary leukostasis and increase in vascular resistance (19). Additionally, stimulated WBCs release some hydrolytic enzymes, cytokines, and growth factors, which have the potential to induce further vascular damage (19). Total leukocyte count enables a low cost, widely available assessment of inflammatory status (19). However, there has been a recent shift away from total WBC count toward differential white cell count as particular WBC types (e.g., neutrophils, lymphocytes, or monocytes) have been suggested to be stronger predictors of cardiovascular risk (48). This is among others because total WBC is influenced by ethnic origin and gender, which is complicating the definition of reference ranges for whole populations (49). An elevated neutrophil count, which is another marker of systemic inflammation, has been suggested as a prognostic marker of cardiovascular disease particularly in hypertensive postmenopausal women (50).

The distribution of WBC subtypes is regulated by the autonomic nervous system since lymphocytes have cholinergic receptors and granulocytes have adrenergic receptors (44, 51). Consequently, the number and function of granulocytes are stimulated by sympathetic nerves, whereby parasympathetic nerves stimulate those of lymphocytes (51). Sympathetic overactivation may be associated with endothelial dysfunction (44).

Mozos et al. found correlations and associations between arterial stiffness and neutrophil monocyte, lymphocyte, and WBC count, respectively, in patients with *hematologic malignancies* and *solid tumors* (52, 53). Increased WBC may be a consequence of the hematologic malignancy, and also a marker of systemic inflammation (47). In chronic granulocytic leukemia, significant associations were found between WBC and arterial stiffness, which are probably related to the increased blood viscosity caused by the high concentration of WBCs (54). Elevated blood viscosity increases vascular shear stress, enabling rapid growth of the atherosclerotic plaque and increasing its instability (55).

Gomez-Sanchez et al. report a positive correlation between central augmentation index (CAIx) and monocyte count only in women, and between neutrophil count and intima-media thickness (IMT) in men, in a study including 500 subjects with *intermediate risk* (56). Gender differences may be explained by higher IMT in men and higher CAIx in women, the influence of sex steroids on vascular function and the influence of anthropometric factors (e.g., differences in the distribution of body fat, average height, as well as aortic length) (56).

In patients with *familial Mediterranean fever* (FMF), which is an autosomal recessive disorder restricted to certain ethnic groups, characterized by recurrent inflammatory febrile attacks of the serosal and synovial membranes; the ongoing and recurring inflammatory state is associated with an increased cardiovascular risk and an elevated arterial stiffness (57, 58). The inflammatory process in FMF results from malfunction of the MEFV gene located on 16p and is associated with uncontrolled inflammatory reactions due to the absence of an inhibitor of C5a and excessive release of tumor necrosis factor and interleukin-1 (57, 59). The MEFV gene encodes mutated protein pyrin, essential in the innate immune system and inflammasome (59). Arterial stiffness (assessed *via* PWV) was correlated with the severity of inflammation and several inflammatory markers, including WBC, CRP, ESR, fibrinogen, and neutrophil/lymphocyte ratio (NLR), and not to the genetic mutation (58). This is in contrast to earlier research where correlations of PWV with leukocyte count but not with CRP in patients with FMF were found (57). This discrepancy might possibly be explainable by a different dosage regime of colchicine of study participants, since colchicine is reported to alter levels of CRP in patients using the drug (60) or confounders that are not known yet or such that were not considered. Sustained inflammation functionally impairs microcirculation, which poses a reason for the increased cardiovascular risk in patients with FMF (58). The increase in the arterial stiffness during attacks might be related to the released cytokines, and subsequent inflammation-induced vasoconstriction (58). Further studies are needed to assess the relationship between arterial stiffness and interleukin-1 and TNF-α in patients with Mediterranean fever.

In conclusion, several studies report a link between arterial stiffness and WBC or leukocyte subtypes, respectively, in particular in patients with hematologic malignancies or FMF, and in postmenopausal women with hypertension (Table 1) (50, 52, 57, 58). It is also important to consider that arterial stiffness modulates the functional responses of WBC, as experimental studies have demonstrated that the mechanical properties of the substrate may influence migration of neutrophils: they migrate more slowly due to significantly larger traction stresses on the stiffer substrates, but more persistently, enabling movement on greater distances over time despite slower speeds (61).

**Table 1:** White blood cell (WBC) count and arterial stiffness.

Study population	Findings	Reference
69 consecutive familial Mediterranean fever (FMF) patients and 35 controls	Pulse wave velocity (PWV) was correlated to serum C-reactive protein (CRP), WBC, erythrocyte sedimentation rate, fibrinogen, and neutrophil/lymphocyte ratio	Cakar et al. (58)
29 patients with hematologic malignancies (8 with multiple myeloma, 2 with Hodgkin’s lymphoma, 11 with non-Hodgkin’s lymphoma, 6 with myelodysplastic syndrome, and 2 with chronic myeloid leukemia)	An inverse correlation was found between PWV and neutrophil count (r = -0.45). Multiple regression analysis found significant associations between augmentation index (AI) and PWV, and WBC and platelet count, respectively	Mozos and Mihaescu (52)
20 patients with solid tumors (colon, lung, renal, laryngeal, pancreas, mammary and testicular cancer, and malignant melanoma) and 26 healthy controls	Significant higher values were obtained for PWV, AI and central hemodynamic variables in cancer patients compared to healthy controls. The best correlation was found between AI and neutrophil count and WBC, respectively. PWV as a measure of arterial stiffness was significantly associated with neutrophilia, monocytosis, and lymphopenia. AI was significantly associated with neutrophilia	Mozos and Mihaescu (53)
500 subjects included in the MARK study, aged 35–74 years	Monocyte count was positively correlated with central augmentation index (CAIx) in women, also after adjusting for age and other confounders	Gomez-Sanchez et al. (56)
886 postmenopausal women with hypertension	A direct relationship between neutrophil count and 24-h ambulatory pulse pressure (PP) was observed. Increased arterial stiffness, as reflected in high values of 24-h ambulatory PP, is an adverse prognostic marker in postmenopausal women with hypertension and can be used as an additional correlate for systemic inflammation	Angeli et al. (50)
23 colchicine-treated patients with FMF and 23 age- and sex-matched controls	PWV was slightly higher in colchicine-treated FMF patients than in control subjects (P = 0.05) and significantly correlated with age and leukocyte count, but no correlations between PWV and CRP levels or blood pressure could be observed PWV was furthermore found to be generally influenced by age and body mass index	Yildiz et al. (57)

Neutrophil/lymphocyte ratio, the ratio between neutrophil and lymphocyte count, is available in routine complete blood count analyses and may be used as a cost-effective biomarker of inflammation, atherosclerotic progression and systemic predictor of cardiovascular complications, especially in context of myocardial infarction and coronary heart disease (19, 62). Chronic low-grade inflammation or subclinical inflammation as indicated by the NLR plays a role in diabetes, obesity, dyslipidemia, hypertension, metabolic syndrome, and endothelial dysfunction (62–64). A higher NLR despite normal WBC shows a higher risk of atherosclerotic disease (65). NLR is a more powerful predictor of cardiovascular disease than any other leukocyte subtype (66). This might be explainable due to the fact that it is less likely to be altered by various physiological conditions (e.g., dehydration or recent exercise) and also, more importantly, that NLR is the ratio of two different, but complementary immune pathways (48).

Inflammation plays an important role in diabetes-induced cardiovascular events related to atherosclerotic injuries. NLR was correlated with aortic stiffness in patients with type 1 diabetes in a study including 76 subjects with type 1 diabetes (19) and in patients with type 2 diabetes mellitus (Table 2) (67).

**Table 2:** Neutrophil/lymphocyte ratio (NLR) and arterial stiffness.

Study population	Findings	Reference
103 patients with STEMI who underwent successful primary percutaneous coronary intervention	Aortic velocity propagation (AVP) values improved after successful treatment in STEMI patients. The increment in AVP values was closely correlated with a decrement in NLR	Yaman et al. (69)
145 consecutive patients admitted with stable angina pectoris or acute coronary syndrome (ACS)	Augmentation index and pulse wave velocity (PWV) were positively associated with NLR. NLR might be used to risk-stratify patients, considering arterial stiffness in patients with coronary heart disease, especially in the presence of ACS	Tanindi et al. (44)
76 persons with type 1 diabetes and 36 healthy controls	The observed significant negative correlation between the NLR and markers of aortic stiffness in patients with type 1 diabetes suggests a strong association between inflammation and arterial stiffness	Ayhan et al. (19)
402 participants:133 control subjects, 138 diabetic subjects without diabetic retinopathy (DR) and 131 patients with DR	NLR and PWV were elevated both in type 2 diabetic and in DR. NLR could be independently associated with PWV	Wang et al. (67)
512 postmenopausal women with a similar socioeconomic background (204 patients with osteoporosis, 208 controls)	NLR and PWV were elevated in osteoporosis. Statistical analysis of collected data revealed a significant correlation between NLR and PWV after adjusting for confounders in osteoporosis patients, but not in the control group	Yu et al. (66)
52 autosomal dominant polycystic kidney disease patients and 25 controls	Pentraxin 3 and C-reactive protein were not correlated with arterial elasticity, while NLR was significantly negatively correlated with large artery elasticity index	Gul et al. (75)
849 Korean adults in a health examination program	A higher NLR was independently associated with arterial stiffness and coronary calcium score	Park et al. (73)

*Coronary heart disease* was associated with elevations in inflammatory markers and changes in leukocyte subset distribution (68, 69). Neutrophil count and NLR are promising markers of the presence and severity of coronary heart disease (70, 71). Endothelial dysfunction in coronary arteries may result from the neutrophil–endothelium interaction, and the increased neutrophil count may accelerate endothelial abnormalities (69, 72). A higher NLR was independently associated with arterial stiffness and coronary calcium score in a large study including 849 Korean adults, revealing that higher NLR may be a useful additional measure for assessing cardiovascular risks (73). Yaman et al. evaluated 103 patients, without a previous history of coronary artery disease (CAD), who presented with STEMI without hemodynamic compromise and underwent successful primary percutaneous coronary intervention. The authors report improvement of arterial stiffness associated with a decrease in NLR (69).

In a recent study, NLR has also been correlated with coronary artery calcium score, which is an independent risk factor for coronary artery stenosis, in asymptomatic Korean males further underlining the high predictive value of NLR in the context of arterial stiffening (62). The beta-blocker nebivolol, which is known to possess anti-inflammatory effects mediated through increased NO-release in ECs, significantly lowered NLR in hypertensive patients (74). NLR has also been associated with arterial stiffness and high coronary calcium score in several previous studies (48). Early detection of abnormal NLR levels may be helpful for detecting increased arterial stiffness in patients with coronary heart disease, type 1 and 2 diabetes mellitus, osteoporosis, and polycystic kidney disease (Table 2) (19, 44, 66, 67, 69, 75).

## Cytokines and Arterial Stiffness

Interleukins (ILs) are a group of cytokines produced by cells of the immune system and have a pro-inflammatory effect. They trigger the acute phase reaction by continued recruitment and activation of leukocytes, and stimulate proliferation of fibroblasts (28). An increased sympathetic tone is associated with higher oxygen consumption and enhanced production of pro-inflammatory cytokines (19, 76). IL-6, IL-1, and TNF-α impair the subendothelial release of NO and increase endothelin-1 release by ECs in a dose-dependent fashion and are thus contributing to the regulation of the vascular tone (19, 77). Endothelin-1 is both a potent vasoconstrictor and mitogen for smooth muscle cells and fibroblasts and IL-6 and IL-1 were found to be the most potent and least effective stimulators of endothelin-1-release, respectively (77). Conflicting results have been obtained on the relationship IL-6-endothelial function by Cotie et al., who found that circulating IL-6 does not mediate endothelial dysfunction, considering that no relationship was observed between IL-6 levels and flow-mediated dilatation (FMD). The authors hypothesized that until there is an overt systemic inflammatory signal, such as in a disease state, no relationship exists (78). Traditional cardiovascular risk factors impair FMD, and Vita et al. found no evidence that inflammation has additional effects beyond those attributable to traditional risk factors (15). Furthermore, cytokines stimulate VSMC and interstitial cell proliferation, contributing to the development of proliferative vascular disorders (77).

Women with a history of preeclamptic pregnancies have an increased risk of cardiovascular disease, but in a recent study no relationship was found between soluble tumor necrosis factor receptor type 1 and other systemic and vascular inflammatory markers increased during *preeclamptic pregnancies* and systemic arterial properties 6 months post-partum, although those markers were increased also at term. Even at term, no general correlation between the increase of systemic and vascular inflammatory markers and systemic arterial properties could be established (79). *Metabolic syndrome*, comprising abdominal obesity, insulin resistance, dyslipidemia, and hypertension, is accompanied by abnormal regulation of cytokines and chemokines, which further underlines the importance of inflammation in the mentioned syndrome (80, 81). Higher plasma levels of IL-6 could be linked to the development of arterial stiffness and microvascular dysfunction (82).

Systemic inflammatory diseases are associated with an increased cardiovascular morbidity and mortality due to endothelial dysfunction and accelerated atherosclerosis (27). Arterial stiffness increases in accelerated atherosclerosis due to inflammation (27). An inflammatory-metabolic background is linked with increased arterial stiffness in patients with seronegative *spondyloarthritis* (SpA), as a positive correlation between PWV, augmentation indices, and ILs are reported in a single study with 108 subjects (53 patients and 55 controls) (83).

IL-18 has been previously associated with the formation, growth, progression, and vulnerability of the atherosclerotic plaque and was identified as an independent predictor of coronary events (84). Although elevated IL-12 and IL-18 levels were not associated with arterial stiffness in patients with *chronic kidney disease* (85), IL-18 was significantly associated with arterial stiffness in patients with metabolic syndrome (86). It is hypothesized that inflammation and arterial stiffness act together in the pathogenesis and complications of metabolic syndrome and type 2 diabetes (86). The lack of association between IL-12 and IL-18 in several studies might be explained by the different effects of inflammation on large and small vessels (85).

Associations between inflammatory gene polymorphism and cardio-ankle vascular index were found only for the cluster of differentiation 14 (CD14) polymorphism among men aged 34–49 years in a study examining polymorphisms in several inflammatory genes (87). CD14 acts as a trigger in the production of cytokines (87). Targeted deletion of genes related to costimulatory factors and pro-inflammatory cytokines results in less atherosclerosis in mouse models, while interference with regulatory immunity accelerates it (30). Among other observed effects that might be beneficial in treatment and prevention of arterial stiffness, vitamin K was also shown in rat studies to suppress inflammation by decreasing expression of genes for cytokines that are associated with arterial stiffness (8, 88).

In summary, ILs and other cytokines have been associated with arterial stiffness in several disorders, such as metabolic syndrome, SpA, and preeclampsia (Table 3) (79, 82, 83).

**Table 3:** Cytokines and arterial stiffness.

Study population	Findings	Reference
34 women with preeclampsia and 61 women with normal pregnancies	Despite increased circulating levels of systemic and vascular inflammatory markers, such as soluble tumor necrosis factor receptor type 1, monocyte chemoattractant peptide 1 pentraxin 3, and soluble vascular adhesion molecule-1 in preeclamptic pregnancies, they are not associated with proximal aortic stiffness and effective arterial elastance	Estensen et al. (79)
53 patients with spondyloarthritis (SpA) and 55 control subjects	Higher mean pulse wave velocity (PWV) and augmentation indices were obtained in patients with seronegative SpA compared to controls. Mean plasma levels of interleukin-6 (IL-6), IL-1β, and TNFα were higher in subjects with elevated PWV. Multivariate analysis revealed a significant association elevated PWV and plasma levels of IL-6, IL-1β, and tumor necrosis factor-α	Tuttolomondo et al. (83)
69 healthy volunteers, 70 chronic kidney disease (CKD) patients stage 3–4, 85 CKD stage 5	IL-12 and IL-18 were found to be elevated during the earlier stages of CKD but could NOT be associated with arterial stiffness	Yong et al. (85)
563 men, aged 64–76 years	Pulse wave propagation time of the brachial artery was independently associated with IL-18	Troseid et al. (86)

## CRP, High-Sensitivity C-Reactive Protein (hsCRP), and Arterial Stiffness

C-reactive protein is an acute phase reactant that predicts cardiovascular events in healthy subjects and in patients with preexisting cardiovascular disorders (89, 90). CRP is used as a marker of chronic low-grade inflammation as it is considered as a mediator of atherothrombotic disease (43, 91, 92) and is the only circulating biomarker related to vascular wall biology (10). CRP was correlated with FMD in some studies (93, 94), but no correlation was found for CRP with coronary endothelial dysfunction in patients with familial hypercholesterolemia (95, 96).

The level of CRP has been associated with indices of arterial function in several populations (7, 17, 21, 80, 97–100). However, data regarding a possible direct etiological role of CRP in arterial dysfunction and atherosclerosis are contradictory, and there are also studies reporting no significant relationship between PWV and hsCRP (17, 101). Tomiyama et al. reported that the association between CRP and arterial stiffness was not retained after adjusting for other cardiovascular risk factors (101).

A significant correlation was reported between aortic flow propagation velocity (AVP), which is a parameter of reduced arterial stiffness, assessed by transthoracic echocardiography, and high CRP levels, which is indicating a link between aortic stiffness and inflammation (7). Several other cross-sectional studies demonstrated the link between metabolic syndrome, arterial stiffness, and inflammation (97). Increased CRP levels were associated with elevated PWV in patients *after renal transplant*, and overall CRP was suggested to be a useful marker to anticipate graft survival and cardiovascular morbidity in renal transplant recipients (102).

Rheumatoid arthritis is a chronic, systemic, inflammatory disease, associated with an increased cardiovascular mortality (21). PWV correlated independently with log-transformed CRP in patients with RA, and immunomodulatory therapy with anti-TNF-α therapy reduced aortic stiffness to levels comparable to those of the healthy control group. This suggests that aortic stiffness may be reversible (21). PWV correlated with current CRP, but not with disease duration, historical inflammation or extent of radiological changes (21). Traditional assays for CRP did not have adequate sensitivity to long term predict vascular disorders, and hsCRP could represent a better predictor. hsCRP was associated with arterial stiffness in several studies (10, 18, 103, 104), but other researchers could not find an association, independent of conventional risk factors (101). Most studies used single measurements of hsCRP instead of a mean of multiple measurements, but a single assessment seems to be adequate if values of less than 10 mg/l are observed (43, 91, 92). hsCRP levels were correlated with traditional cardiovascular risk factors, such as hypertension, dyslipidemia, overweight, and obesity, but also with other inflammatory markers, including WBC, IL-6, and fibrinogen level (10).

Vascular hsCRP production is stimulated by cytokines (IL-6 and IL-1) and has modulatory functions by inhibiting endothelial NO synthase and inducing the expression of adhesion molecules in ECs. hsCRP has also a major role in increasing cytokines expression and generation of reactive oxygen species by monocytes and neutrophils, promoting vasoconstriction, VSMC migration and proliferation, activation of platelets, and vascular stiffness (18, 43, 104). Increased hsCRP may be also a consequence of arterial stiffness because increased arterial stiffness is associated with higher flow reversals during diastole, which can increase the expression of adhesion molecules (43).

C-reactive protein and hsCRP were associated with arterial stiffness in patients with metabolic syndrome, renal transplant, diabetes mellitus, and RA (Table 4) (7, 21, 102, 104). Many interventions able to reduce cardiovascular risk have been associated with lower hsCRP values, such as weight loss, diet, exercise, smoking cessation, use of lipid-lowering drugs (statins, niacin, fibrates, gemfibrozil), aspirin, and thiazolidinediones (92).

**Table 4:** C-reactive protein (CRP), high-sensitivity C-reactive protein (hsCRP), and arterial stiffness.

Study population	Findings	Reference
100 patients with metabolic syndrome, 14 controls	There was a significant correlation between aortic flow propagation velocity (AVP), FMD (flow-mediated dilatation), and high CRP, indicating a possible link between aortic stiffness, endothelial dysfunction, and inflammation	Adel et al. (7)
150 renal transplant recipients	High (> 20 mg/L) post-transplant CRP levels predicted pulse wave velocity (PWV) and cardiovascular morbidity in a two-year time period after renal transplantation	Gurlek Demirci et al. (102)
40 patients with type 2 diabetes mellitus	hsCRP showed a moderate positive correlation with arterial stiffness in patients with type 2 diabetes mellitus	Nurizal et al. (104)
825 men (mean age: 74 years)	Arterial stiffness was found to correlate positively with circulating levels of CRP	McEniery et al. (100)
362 middle-aged and elderly men	Low-grade inflammation was shown to be independently related to increase of aortic artery stiffness over and above traditional risk factors and atherosclerosis	Nakhai-Pour et al. (43)
77 patients with rheumatoid arthritis (RA) and 142 healthy individuals	Median aortic PWV was significantly higher in RA patients than in control subjects and correlated independently with age, mean arterial pressure, and CRP	Mäki-Petäjä et al. (21)
214 asymptomatic subjects with a mean age of 59 years	CRP was related to measures of arterial wave reflection and stiffness in asymptomatic subjects	Kullo et al. (99)
866 participants above 55 years	Levels of CRP were linearly associated with PWV	Mattace-Raso et al. (98)
2,668 Japanese men (43 ± 10 years old)	PWV showed a significant correlation with the logarithm of hsCRP, but multiple linear regression analyses demonstrated that the logarithm of hsCRP was not significantly related to PWV, independent from conventional risk factors	Tomiyama et al. (101)
158 apparently healthy subjects (age range 40–65 years)	Plasma levels of hsCRP were positively correlated with AIx, central pulse pressure and central systolic blood pressure	Kampus et al. (103)

## Cell Adhesion Molecules and Arterial Stiffness

Soluble cell adhesion molecules include intercellular adhesion molecule-1 (ICAM-1), vascular cell adhesion molecule-1 (VCAM-1), the platelet (P-selectin), and endothelial selectin (E-selectin) (34). Adhesion molecules are glycoproteins involved in tissue integrity, mediation of cellular communication and interactions, and extracellular matrix contact (34). They are increased in endothelial dysfunction, vascular remodeling, and obesity. They are furthermore considered as biomarkers and mediators of cardiovascular disorders in several cardiovascular disorders, including hypertension, stroke, and coronary heart disease (34, 105, 106). They accelerate atherosclerosis by enabling attachment of circulating leukocytes to ECs (105). E-selectin and P-selectin mediate transient rolling of leukocytes along the endothelium and ICAM-1 and VCAM-1 mediate stronger attachment of leukocytes to the endothelium (106). Adhesion molecules can not only be detected on the endothelial surface but also as soluble adhesion molecules in the circulation, where they are reported as useful biomarkers to predict future fatal cardiovascular events in patients with angiographically documented CAD (107).

Selectins are C-type lectins, including L-selectin expressed on leukocytes, E-selectin expressed by cytokine-activated ECs and P-selectin which is expressed by platelets and ECs (108). E-selectin is produced exclusively by ECs and is therefore considered a superior marker of endothelial dysfunction compared to the other cell adhesion molecules, while ICAM-1 and VCAM-1 are expressed on both ECs and leukocytes (106). ICAM-1 is also expressed in hematopoietic cells and fibroblasts and was suggested to be used as a marker of low-grade inflammation (109). VCAM-1 may be a marker of plaque activity (109), but elevated VCAM-1 levels could have a protective effect from cardiovascular events in the general population (106). While Kilic et al. found no correlation between VCAM-1 and ICAM-1 and arterial stiffness, de Faria et al. reported higher VCAM-1 values in patients with increased arterial stiffness (34, 109). Further studies assessing VCAM-1 and arterial stiffness could clarify the relationship between VCAM-1 and arterial stiffness.

Kals et al. enrolled 39 patients with *peripheral arterial disease* and 34 controls and found a significantly reduced endothelial function index, an increased augmentation index (AI), estimated PWV, ICAM-1, hsCRP, myeloperoxidase, and urinary 8-iso-prostaglandin F2a (110). They found an inverse, significant association between endothelial function index and ICAM-1 only in the controls, and significant correlations between PWV and AI, respectively, and urinary 8-iso-prostaglandin F2a in patients (110). The study demonstrated the importance of the degree of inflammation for endothelial vasomotor capacity in the subclinical condition and of oxidative stress for arterial stiffness (110).

Vascular adhesion protein-1 (VAP-1) is associated with cell membranes (in ECs, smooth muscle cells, and adipocytes) but also found as soluble VAP-1 in plasma. It serves both the function as an adhesion molecule and an amine oxidase producing aldehyde and hydrogen peroxide. It is involved in vascular injury due to its semicarbazide-sensitive amine oxidase (SSAO) activity because of releasing of formaldehyde and methylglyoxal from the breakdown of primary amines, which is responsible for direct cytotoxic damage to ECs (111, 112). VAP-1 was associated with arterial stiffness in *subjects over 60 years* after adjusting for PWV-related confounders (112). Inflammation is the main link between VAP-1 and arterial stiffness, impairing the balance between production and degradation of collagen and elastin fibers, resulting in overproduction of abnormal collagen and reducing quantities of normal elastin (6). As an adhesion molecule, VAP-1 is involved in the rolling, adhesion, and transmigration of lymphocytes, granulocytes, and monocytes from the blood into the vessel wall, and the oxidase activity of VAP-1 may have signaling effects and induce expression of E- and P-selectins and ICAM-1 in ECs (113, 114). VAP-1 is also involved in endothelial dysfunction and synthesis of advanced glycation end products that are well known to contribute to an increase in arterial stiffness. Furthermore, SSAO activity was associated with an abnormal elastin structure and production of reactive oxygen species (112).

In *systemic lupus erythematosus*, antiphospholipid antibodies bind to receptors on the endothelium to upregulate adhesion molecules such as E-selectin, ICAM-1, and VCAM-1, but the presence of the antibodies was not associated with arterial stiffness (27).

In summary, adhesion molecules were associated with arterial stiffness in patients with a peripheral arterial disease, resistant hypertension, and subjects over 60 years (Table 5) (34, 110).

**Table 5:** Adhesion molecules and arterial stiffness.

Study population	Findings	Reference
110 patients with resistant hypertension and 112 mild to moderate hypertensive patients	sP-selectin and sVCAM-1 were elevated in the presence of arterial stiffness and cardiac hypertrophy	de Faria et al. (34)
63 participants referred for echocardiography	No correlation was demonstrated between indices of aortic stiffness and vascular cell adhesion molecule-1 and intercellular adhesion molecule-1 levels	Kilic et al. (109)
568 Han Chinese healthy persons with an age 30 or older	Plasma vascular adhesion protein-1 (VAP-1) was associated with arterial stiffness in older individuals. VAP-1 may be important for vascular aging	Chen et al. (112)
39 patients with peripheral arterial disease and 34 controls	There was an inverse association between endothelial function index and intercellular adhesion molecule-1 in the controls, but not in the patients	Kals et al. (110)

## Oral Infections and Arterial Stiffness

The association between oral inflammation and the risk of major cardiovascular events, such as myocardial infarction and stroke, was described two decades ago (115, 116). Bacteria and their products from the dental plaque and crevicular fluid are involved in local destruction of gingiva and bone and they are also released to the bloodstream (117).

Periodontitis is associated with endothelial dysfunction due to periodontal pathogens, atherosclerosis, an increased risk of myocardial infarction, stroke, and peripheral arterial disease (118, 119). A 19% increase in the risk of cardiovascular disease was reported, and the risk increases in elderly patients (119, 120). Periodontitis and cardiovascular disorders share several risk factors, such as age, heredity, smoking, diabetes mellitus, hypertension, estrogen deficiency in women, a low socioeconomic status, and stress (117, 119).

Microbial pathogens associated with periodontal disease, predominantly Gram-negative bacteria, cause high levels of bacteremia after routine dental procedures and every day activities, including tooth brushing (121) and a chronic, progressive, destructive, and unresolved inflammation (122, 123). Periodontal pathogens and their noxious products gain access to the periodontal tissues and are released into the systemic circulation through the ulcerated sulcular epithelium of the gingiva, and they are a source of inflammatory mediators, which can cause insulin resistance, systemic inflammation, and explain, probably, the perio-systemic link (119, 124, 125). *Porphyromonas gingivalis* and *Treponema denticola*, bacteria causing periodontal disease, have been found in the atherosclerotic plaque (117). The levels of biomarkers such as hsCRP, TNF-α, and interleukin-6 and -1 were increased in patients with periodontitis. The autoimmune mechanism is involved in both periodontal disease and RA, which explains the significant association of both disorders associated with an increased cardiovascular risk (119). High periodontal bacteria antibodies titers were associated with atherosclerosis, regardless of smoking status (118, 126), and improvement in clinical and microbial periodontal status was associated with a decreased rate of carotid artery IMT progression (127). Probably, the atherosclerotic process is initiated or accelerated by the inflammatory periodontal reaction (128). It is also possible that a common pathway leads independently to both periodontal disease and atherosclerosis (117).

Several studies reported an increased arterial stiffness in patients with periodontitis compared to controls, suggesting that patients with periodontitis suffer from a subclinical vascular dysfunction (Table 6) (129–134). Future research should point out the relationship between active treatment of periodontitis and cardiovascular benefits, because the results of the interventional studies are contradictory, especially in obese patients (117, 133, 135).

**Table 6:** Periodontitis and arterial stiffness.

Study population	Findings	Reference
80 volunteers and 33 pairs of periodontitis patients, matched by age and gender	An association between arterial stiffness and periodontitis was suggested by a lower degree of uniformity in the transmission of the pulse wave through the carotid arteries	Sanz-Miralles et al. (136)
57 periodontitis patients and 48 healthy controls	PWV was measured and followed up 6 months after periodontal therapy. PWV was significantly higher in periodontitis patients compared to the reference group even after adjusting for cardiovascular risk factors. Periodontal treatment did not lower significantly PWV	Houcken et al. (134)
92 patients with periodontitis and 66 matched healthy controls	Significantly higher PWV, aortic augmentation index, and central blood pressure and lower pulse pressure amplification values were found in subjects with severe or aggressive periodontitis	Jockel-Schneider et al. (132)
273 indigenous Australian adults	Arterial stiffness significantly increased with increasing extent of periodontal pocketing	Kapellas et al. (131)
532 gingivitis and 282 periodontitis cases (Asian Indians)	PWV and arterial stiffness index were elevated in periodontitis compared to gingivitis cases and in those with diabetes and hypertension	Shanker et al. (130)
1,053 Japanese subjects with 10 teeth or more	A linear, dose-dependent relationship was found between periodontal pocket depth and arterial stiffness	Hayashida et al. (129)
26 patients with refractory hypertension and generalized chronic periodontitis	Periodontal therapy significantly reduced the level of CRP, IL-6, fibrinogen, blood pressure, left ventricular mass and arterial stiffness, lowering cardiovascular risk	Vidal et al. (135)
16 periodontally healthy, 87 gingivitis, and 18 periodontitis patients with type 2 diabetes mellitus	Periodontal inflammation in patients with type 2 diabetes mellitus is associated with increased intima-media thickness and blood pressure, but not with greater arterial stiffness	Franek et al. (117)

*PWV, pulse wave velocity; CRP, C-reactive proteins; IL-6, interleukin-6*.

Concluding, arterial stiffness is impaired in patients with periodontitis. No study reported yet a symbiotic relationship between periodontitis and arterial stiffness or any relationship between odontogenic foci and arterial stiffness, and those could be aims of future studies.

## MicroRNAs and Vascular Inflammation

Increasing evidence indicate that miRNAs, which are small non-coding RNAs, able to regulate gene expression and to prevent or reduce protein synthesis, have distinct profiles and play crucial roles in various physiological and pathological processes, including vascular aging and inflammation (137–140). An imbalance in the normal miRNA profile can be identified long before the onset of a disease (139, 141). MicroRNAs, such as miR-126 and miR-10a, can control vascular inflammation due to leukocyte activation and infiltration through the vascular wall (142). MicroRNA-663 specifically mediated shear stress-induced monocyte adhesion to ECs (143). Let-7g reduced, besides ECs senescence, also EC inflammation and monocyte adhesion (144). MicroRNA-181b shift macrophage polarization toward M2 anti-inflammatory phenotype, reducing macrophage accumulation and enabling tissue repair (145). Another microRNA, miR-92a, the “atheromiR candidate,” upregulated by oxLDL, is a pro-inflammatory regulator in ECs by modulating inflammatory cytokines and chemokines, enabling monocyte adhesion (146). MicroRNA-92a controls also neovascularization after ischemic injury (140). MicroRNAs can also regulate adhesion molecules expression, especially miR-21 and miR-126. MicroRNA-21, highly expressed in the cardiovascular system, is the most abundant microRNA in monocytes/macrophages, related to a pro-inflammatory phenotype, control of the flow-induced inflammatory response, mediating the balance of pro- and anti-inflammatory responses, expression of adhesion molecules, polarization of macrophages and macrophage apoptosis (147). Zhou et al. demonstrated that miR-21 acts as an epigenetic mediator of the pro-inflammatory phenotype of the vascular ECs exposed to oscillatory shear stress, inhibiting expression of peroxisome proliferators-activated receptor-alpha and upregulating activator protein-1 and expression of adhesion molecules (138). A study including 95 hypertensive patients, evaluated at baseline and after 1 year of effective antihypertensive therapy, showed independent correlations between levels of miR-21 and changes of PWV, independent of blood pressure values, highlighting the significance of miR-21 in vascular remodeling (139). MicroRNA-21 enables also fibrosis in the vascular wall related to AII, promoting proliferation of interstitial fibroblasts and deposition of the extracellular matrix (139). It is also involved in regulation of VSMC phenotype and suppression of endothelial progenitor cell proliferation (139). Harris et al. showed that miR-126 inhibits VCAM-1 expression, limiting leukocyte adhesion to the vascular endothelium (137). However, miR-126 is also involved in angiogenic signaling (141). MicroRNA-30 reduces basal and TNF-α-induced expression of adhesion molecules: VCAM-1, ICAM-1, and E-selectin, impairing the expression of angiopoietin 2 and contributing to the atheroprotective effects of shear stress (148). Deng et al. enrolled 406 Chinese participants in a study and measured miR-1185, adhesion molecules levels (VCAM-1 and E-selectin), and arterial stiffness, reporting independent correlations of microRNA with PWV and adhesion molecules, respectively (149).

It is too early to consider microRNAs as diagnostic and prognostic biomarkers or therapeutic target for arterial stiffness, because there are very few studies linking vascular function and non-coding RNAs. However, miRNAs could be useful as markers of vascular remodeling and plaque instability in future studies.

## Cyclooxygenase-2 (COX-2), Prostaglandin Signaling, and Arterial Stiffness

During inflammation, phospholipase brakes down phospholipids of the WBC membrane into arachidonic acid, which enables synthesis of prostaglandins through the cyclooxigenase pathway, involving cyclooxygenase 1 and 2. The prostaglandins formed by the COX-2 pathway perpetuate inflammation and amplify the effects of other inflammatory mediators, due to its upregulation in monocyte-derived macrophages from the atherosclerotic lesions (150, 151). Microsomal prostaglandin E2 synthase-1 (mPGES-1) catalyzes prostaglandin E2 generation from prostaglandin H2, is primary coupled to COX-2, is strongly upregulated in inflamed tissues, and represents a key enzyme in atherosclerosis and stroke (152, 153). In atherosclerosis of the carotid arteries, both COX-2 and mPGES-1 are upregulated in the vulnerable area of the plaque and may favor plaque instability (152).

The relationship between COX-2 and vascular remodeling was described in the pulmonary circulation. Pulmonary arterial stiffness occurs early in experimental pulmonary hypertension and is an independent risk factor for mortality in pulmonary hypertension (154). COX-2 is upregulated in pulmonary artery smooth muscle cells during hypoxia, associated with upregulation of the endothelin receptor (155) and intravascular macrophage accumulation (156). The transcriptional regulator Yes-associated protein activity is increased in pulmonary hypertension in pulmonary artery smooth muscle cells and is necessary for development of stiffness-dependent remodeling phenotypes (154). However, selective COX-2 inhibition impairs the balance of vascular mediators, enables vascular hyperplasia, remodeling of the systemic vessels, platelet deposition, intravascular thrombosis, and is associated with several cardiovascular events (155).

Cyclooxygenase-2 derived prostaglandin E2, acting on EP1 receptors, is responsible for increased arterial stiffness, increased vasoconstrictor responses, extracellular matrix deposition, endothelial dysfunction, and vascular inflammation (157). AII induces expression of COX-2 and prostanoids in several vessels, and high levels of AII are expressed in vessels of hypertensive patients (157).

On the other hand, Vlachopoulos et al. demonstrated that, especially, COX-1, and also COX-2 mediate the unfavorable effects of smoking on arterial stiffness (158). Endothelial function was not improved by indomethacin or rofecoxib in patients with RA (159), and meanwhile, rofecoxib was withdrawn from the market considering the increased risk of atherothrombotic events associated with this class of drugs (150).

Despite the mechanisms linking COX-2, inflammation, and arterial stiffness, chronic COX-2 inhibition is not an effective destiffening solution, because of its prothrombotic and hypertensive undesirable effects. There is urgent need for the discovery of new effective and cardiovascular safe anti-inflammatory drugs, and mPGES-1 inhibitors may be the solution.

## Study Limitations

Most of the studies were cross-sectional, often with only a relatively small number of participants, and sometimes with just one measurement of inflammatory markers, all of which is unfavorable for the significance of results and their interpretation. The cross-sectional design of most studies cannot establish causality, it can only establish an association (102). Relatively small numbers of study participants, which are often a specific subgroup of patients with certain morbidities does not allow to extend conclusions to the general population; therefore, the results of many studies presented here need confirmation in larger studies. Other limitations arise from an election bias due to exclusion of patients with active infections, and not considering PP amplification and the fact that arterial stiffness is not a uniform condition in all arteries (19, 98). Also, the possibility of undetected disease in study participants limits the significance of studies (57). Usually, patients with established and not early disease were studied and it is not clear if changes in arterial properties precede increase of inflammatory markers and if there is an etiological link between inflammatory markers and cardiovascular complications. It is also still not clear if old or cumulative inflammation or just acute or subacute inflammation correlate with arterial stiffness (21).

Several confounding factors may influence the level of inflammatory markers and not all of them are known currently. The level of soluble adhesion molecules is, for example, at least influenced by age, smoking status, diabetes mellitus, other inflammatory conditions, exercise, and changes in blood pressure (109). The variance of CRP due to LDL cholesterol was less than 3–5%, and values of CRP are stable over long periods, with almost no circadian variation and are not influenced by food intake (92). Major infections, trauma, and hospitalization may increase CRP 100-fold or more, and values exceeding 10 mg/l should be ignored and the test should be repeated (92).

Also, the methodologies of assessing arterial stiffness are limiting the significance of the obtained results. For example, PWV measurements have the immanent problem that it remains difficult to accurately record the femoral pressure wave in participants with peripheral artery disease, and also obesity affects the absolute value of PWV by overestimating the distance and thus yielding artificial values (160). Also, measurements of blood pressure values present a possible source of limiting significance, since blood pressure values are often derived *via* the brachial PP using a sphygmomanometer and are then converted into central PP by a transfer function. However, since vascular dimensions depend on body size and vascular properties vary with arterial pressure, age, or treatment, the premise that the characteristics of the vascular system between the two measuring sites are the same in all individuals and under all conditions is therefore not true (161). More sophisticated inflammatory markers, such as adhesion molecules and ILs, are inappropriate for routine clinical use (91, 92).

## Conclusion

There is ample evidence of a crosstalk between arterial stiffness and systemic inflammation, and inflammation plays an important role in the development of arterial stiffness. Inflammatory markers may be useful additional tools in the assessment of the cardiovascular risk, atherosclerotic plaque remodeling, and preclinical atherosclerotic changes in clinical practice and may be used to develop risk scores for possible future cardiovascular events. This might help to close the currently existing gap between predicted cardiovascular events and their real prevalence. Most of the inflammatory markers are inexpensive and easily measurable, widely available, standardized and may be included in the annual examination of patients at risk, considering that inflammation causes a reversible increase of arterial stiffness.

Several studies revealed significant associations between arterial stiffness and inflammatory markers, such as WBC, NLR, adhesion molecules, fibrinogen, CRP, hsCRP, cytokines, microRNAs, and COX-2 in patients with metabolic syndrome, diabetes mellitus, coronary heart disease, systemic and pulmonary hypertension, peripheral arterial disease, malignant and inflammatory rheumatic disorders, polycystic kidney disease, renal transplant, FMF, in women with preeclampsia or after menopause, and in patients with severe periodontitis. Further studies are needed, in several other disorders and healthy subjects, to confirm the reversible association linking arterial stiffness and inflammatory markers and its predictive value for cardiovascular events.

Combined assessment of arterial stiffness and inflammatory markers may improve non-invasive assessment of cardiovascular risk in several disorders, enabling selection of high-risk patients for prophylactic treatment or more regular medical examination. Development of future destiffening therapies may target pro-inflammatory mechanisms, including miRNAs, enabling stabilization of the atherosclerotic plaque, control of cardiovascular risk factors, and inflamm-aging.

## Author Contributions

IM has written the first draft of the manuscript. CM, JH, CG, DS, CL, and AA revised and improved the first draft. All authors have seen and agreed on the finally submitted version of the manuscript.

## Conflict of Interest Statement

The authors declare that the research was conducted in the absence of any commercial or financial relationships that could be construed as a potential conflict of interest.
